# Percutaneous transhepatic vs. endoscopic retrograde biliary drainage for suspected malignant hilar obstruction: study protocol for a randomized controlled trial

**DOI:** 10.1186/s13063-018-2473-2

**Published:** 2018-02-14

**Authors:** Firas Al-Kawas, Harry Aslanian, John Baillie, Filip Banovac, Jonathan M. Buscaglia, James Buxbaum, Amitabh Chak, Bradford Chong, Gregory A. Coté, Peter V. Draganov, Kulwinder Dua, Valerie Durkalski, B. Joseph Elmunzer, Lydia D. Foster, Timothy B. Gardner, Brian S. Geller, Priya Jamidar, Laith H. Jamil, Rajesh N. Keswani, Mouen A. Khashab, Gabriel D. Lang, Ryan Law, David Lichtenstein, Simon K. Lo, Sean McCarthy, Silvio Melo, Daniel Mullady, Jose Nieto, J. Bayne Selby, Vikesh K. Singh, Rebecca L. Spitzer, Brian Strife, Paul Tarnaksy, Jason R. Taylor, Jeffrey Tokar, Andrew Y. Wang, April Williams, Field Willingham, Patrick Yachimski, In alphabetical order for the INTERCPT Study Group and the United States Cooperative for Outcomes Research in Endoscopy (USCORE)

**Affiliations:** 1 Division of Gastroenterology, Johns Hopkins Sibley Memorial Hospital, Washington, DC USA; 2 Division of Gastroenterology, Yale University, New Haven, CT USA; 3 Division of Gastroenterology, Virginia Commonwealth University, Richmond, VA USA; 4 Division of Interventional Radiology, Vanderbilt University, Nashville, TN USA; 5 Division of Gastroenterology, Stony Brook University, Stony Brook, NY USA; 6 Division of Gastroenterology, University of Southern California, Los Angeles, CA USA; 7 Division of Gastroenterology, Case Western Reserve University, Cleveland, OH USA; 8 Division of Gastroenterology and Hepatology, Medical University of South Carolina, Charleston, SC USA; 9 Division of Gastroenterology, Hepatology, and Nutrition, University of Florida, Gainesville, FL USA; 10 Division of Gastroenterology, Medical College of Wisconsin, Milwaukee, WI USA; 11 Department of Public Health Sciences, Medical University of South Carolina, Charleston, SC USA; 12 Division of Gastroenterology, Dartmouth University, Lebanon, NH USA; 13 Division of Interventional Radiology, University of Florida, Gainesville, FL USA; 14 Division of Gastroenterology, Cedars Sinai Medical Center, Los Angeles, CA USA; 15 Division of Gastroenterology, Northwestern University, Chicago, IL USA; 16 Division of Gastroenterology, Johns Hopkins Medical Institutions, Baltimore, MD USA; 17 Division of Gastroenterology, Washington University, St. Louis, MO USA; 18 Division of Gastroenterology, University of Michigan, Ann Arbor, MI USA; 19 Division of Gastroenterology, Boston University, Boston, MA USA; 20 Division of Gastroenterology, Ohio State University, Columbus, OH USA; 21 Division of Gastroenterology, University of Florida-Jacksonville, Jacksonville, FL USA; 22 The Borland-Groover Clinic, Jacksonville, FL USA; 23 Division of Interventional Radiology, Medical University of South Carolina, Charleston, SC USA; 24 Division of Interventional Radiology, Virginia Commonwealth University, Richmond, VA USA; 25 Division of Gastroenterology, Methodist Dallas Medical Center, Dallas, TX USA; 26 Division of Gastroenterology, Saint Louis University, St. Louis, MO USA; 27 Division of Gastroenterology, Fox Chase Cancer Center, Philadelphia, PA USA; 28 Division of Gastroenterology, University of Virginia, Charlottesville, VA USA; 29 Division of Gastroenterology, Emory University, Atlanta, GA USA; 30 Division of Gastroenterology, Vanderbilt University, Nashville, TN USA

**Keywords:** Cholangiocarcinoma, Hilar stricture, Endoscopic retrograde cholangiopancreatography, Percutaneous transhepatic biliary drainage

## Abstract

**Background:**

The optimal approach to the drainage of malignant obstruction at the liver hilum remains uncertain. We aim to compare percutaneous transhepatic biliary drainage (PTBD) to endoscopic retrograde cholangiography (ERC) as the first intervention in patients with cholestasis due to suspected malignant hilar obstruction (MHO).

**Methods:**

The INTERCPT trial is a multi-center, comparative effectiveness, randomized, superiority trial of PTBD vs. ERC for decompression of suspected MHO. One hundred and eighty-four eligible patients across medical centers in the United States, who provide informed consent, will be randomly assigned in 1:1 fashion via a web-based electronic randomization system to either ERC or PTBD as the initial drainage and, if indicated, diagnostic procedure. All subsequent clinical interventions, including crossover to the alternative procedure, will be dictated by treating physicians per usual clinical care. Enrolled subjects will be assessed for successful biliary drainage (primary outcome measure), adequate tissue diagnosis, adverse events, the need for additional procedures, hospitalizations, and oncological outcomes over a 6-month follow-up period. Subjects, treating clinicians and outcome assessors will not be blinded.

**Discussion:**

The INTERCPT trial is designed to determine whether PTBD or ERC is the better initial approach when managing a patient with suspected MHO, a common clinical dilemma that has never been investigated in a randomized trial.

**Trial registration:**

ClinicalTrials.gov, Identifier: NCT03172832. Registered on 1 June 2017.

**Electronic supplementary material:**

The online version of this article (10.1186/s13063-018-2473-2) contains supplementary material, which is available to authorized users.

## Background

Both percutaneous transhepatic biliary drainage (PTBD) and endoscopic retrograde cholangiography (ERC) are accepted approaches in the management of patients with malignant obstruction at the liver hilum. In routine clinical practice, ERC is generally favored on the basis of: (1) high technical and clinical success rates for other (non-hilar) indications; (2) the perceived safety of ERC relative to PTBD; (3) the perceived ability to perform more comprehensive tissue sampling at the time of ERC compared to PTBD; (4) the avoidance of external tubes which are often needed for PTBD; and (5) because patients with suspected malignant hilar obstruction (MHO) typically present to, and are managed by, gastroenterologists.

However: (1) observational data suggest that PTBD is superior for achieving complete drainage of MHO [1–3] and some guidelines recommend the percutaneous approach over ERC for Bismuth type 3 and 4 hilar strictures [4]; (2) the generally quoted risks of PTBD are based on outdated studies and may be exaggerated [5]; and (3) endoscopic diagnosis of indeterminate biliary strictures remains suboptimal despite the use of cholangioscopy and multi-modal sampling.

Although existing evidence suggests that many patients who undergo initial ERC require subsequent PTBD for adequate drainage [3, 6], no randomized trials comparing the two modalities for suspected MHO have been published. We hypothesize that even though PTBD will be more effective than ERC for decompression of suspected MHO, this advantage will be offset by the favorable safety profile and superior diagnostic capacity of ERC. If, however, PTBD is found to be substantially superior in terms of drainage, or if the potential advantages of ERC are not realized, then the existing clinical approach to MHO must be reappraised. Moreover, identifying patient and stricture characteristics that predict response to PTBD or ERC may be important for informing clinical decision-making and guidelines.

## Methods

### Design

The INTerventional Radiology vs. ERC for Perihilar Tumors (INTERCPT) trial is a multi-center, comparative effectiveness, randomized, superiority trial of PTBD vs. ERC for decompression of suspected MHO. Ethical approval has been obtained from the Institutional Review Board at the primary site (Medical University of South Carolina, Pro00063825) and several sub-sites. The remaining sites will only enroll subjects once local regulatory approval has been obtained.

### Patients

The eligibility criteria are listed in Table 1. Informed consent will be obtained from all study participants. We plan to enroll patients of 40 years of age or older with cholestasis who have radiographic evidence of obstruction at the liver hilum. Thus, patients with a documented hilar stricture on magnetic resonance imaging (MRI) or those with a dilated intrahepatic, but not extrahepatic biliary system, on ultrasound or computed tomography scan (CT) will be eligible. To maximize generalizability, this trial is intended to mimic routine clinical practice as closely as possible, in which the decision to proceed with biliary decompression is often based on information from one – but not all – of these radiographic tests. Patients will be excluded if the hilum is not directly involved, if there is reason to believe that the stricture is benign (because these are typically better managed endoscopically or surgically), or if there are relative contraindications to PTBD or ERC that clearly favor one procedure over the other.

**Table 1:** Eligibility criteria

Inclusion criteria
1. Age ≥ 40 years (to reduce the likelihood of enrolling patients with obstruction due to primary sclerosing cholangitis)
2. Cholestatic liver function tests, including serum alkaline phosphatase level ≥ 300 IU/L and bilirubin level ≥ 3.7 mg/dL
3. Radiographic evidence of a biliary hilar stricture *or* intrahepatic *but no extrahepatic* biliary ductal dilation
Exclusion criteria
1. Known radiographic evidence of a Bismuth-Corlette type 1 biliary stricture
2. Known diagnosis of primary sclerosing cholangitis *without* suspicion of dominant hilar stricture
3. Recent cholecystectomy, liver resection, or biliary surgery within 12 months
4. Known Mirizzi syndrome
5. Known IgG4-mediated cholangiopathy
6. Significant liver metastatic disease interfering with safe/effective PTBD
7. Significant ascites interfering with safe/effective PTBD
8. Known regional malignant-appearing adenopathy or extrabiliary mass, indicating the need for concurrent EUS-FNA
9. Prior ERC or PTBD for hilar obstruction
10. Surgically altered luminal anatomy other than prior Billroth reconstruction
11. Standard general contraindications to ERC or PTBD (e.g., hemodynamic instability, uncorrected coagulopathy, etc.)
12. Pregnancy
13. Inability or unwillingness to follow study protocol

*ERC,* endoscopic retrograde cholangiography, *EUS-FNA*, endoscopic ultrasound-guided fine-needle aspiration biopsy, *PTBD* percutaneous transhepatic biliary drainage

### Setting

Patients will be identified and enrolled at approximately 25 tertiary medical centers across the United States, the exact setting in which such patients are managed in routine clinical practice and the decision of PTBD vs. ERC made because their high complexity generally prompts referral to a tertiary center. Non-referral hospitals were not included in the network because MHO patients require not only tertiary and quaternary procedural care (to include interventions like cholangioscopy, intraductal photodynamic therapy, and radiofrequency ablation), but also National Cancer Institute (NCI)-designated cancer center-type expertise in medical and surgical oncology as part of a multi-disciplinary Tumor Board approach.

### Randomization

**Fig. 1:**
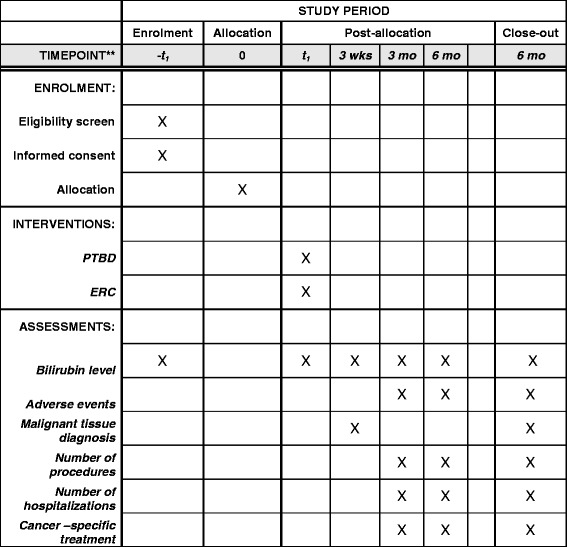
Enrollment, intervention, and assessments in the INTERCPT trial

Eligible patients who provide informed consent will be randomly assigned in 1:1 fashion to PTBD or ERC as the first intervention using a web-based, electronic randomization system. The randomization schedule will be generated centrally at the data coordinating center and will ensure treatment balance within site. The study allocation, interventions, and assessments as adapted from the Standard Protocol Items: Recommendations for Interventional Trials (SPIRIT) Figure, are outlined in Fig. 1; Additional files 1 and 2.

### Procedure

Enrolled subjects will undergo PTBD or ERC according to study group assignment. All components of the procedure and related interventions will be dictated by treating physicians per usual care. Specifically, the technical approach to drainage (e.g., bilateral vs. unilateral, functional lobe only) and tissue sampling (e.g. brushing, intraductal biopsies, cholangioscopy, fluorescence in situ hybridization, etc.) will be determined by the treating physicians and proceduralists performing the PTBD or ERC.

### Blinding

Given the substantial differences in approach between PTBD and ERC, including pre-procedure instructions and consent, post-procedure observation and follow-up, location within a hospital, as well as the need for a percutaneous catheter after PTBD, blinding subjects to study intervention is not possible. Similarly, there is no practical method to ensure masking of treating clinicians and outcome assessors who will be reviewing medical records.

### Follow-up

All follow-up decisions pertaining to the need for repeat procedures to address inadequate drainage or incomplete tissue sampling, or the decision to refer the patient for the alternative procedure will be dictated by treating physicians. All enrolled subjects will be followed for 6 months after the index (randomly assigned) procedure, primarily through review of their medical records.

### Outcomes

The primary endpoint of successful biliary drainage will be defined as a 50% reduction in bilirubin level within 3 weeks of the study intervention without additional ERC or PTBD during that timeframe.

Secondary endpoints will include:

1. An alternative definition of successful biliary drainage, defined as improvement in the serum bilirubin level to ≤ 2.5 mg/dL as a result of the index (randomization) intervention without the need for additional PTBD or ERC. This bilirubin level is considered informative because it is the threshold most commonly used by oncologists to administer chemotherapy
2. Adverse events related to PTBD and ERC, defined according to standard consensus guideline documents published in the interventional radiology [5] and gastroenterology [7] literature, respectively
3. Malignant tissue diagnosis, defined as a definitive diagnosis of malignancy documented in the subject’s medical record. We recognize that a fraction of patients will not have malignant obstruction, will not require a tissue diagnosis (known alternative primary cancer), or that a “gold standard” diagnostic test may not be available within the follow-up period in patients who are not diagnosed with cancer. We do, however, expect that patients with cancer in whom a tissue diagnosis is needed and possible will be allocated evenly between study groups
4. Total number of procedures, hospitalizations, and cancer-specific treatments during the 6-month follow-up period

### Statistical considerations

Given patient preference to avoid extracorporeal tubes as well as the other perceived advantages of ERC, we estimate that PTBD would have to be at least 20% more effective in achieving successful biliary drainage to change clinical practice. Assuming that PTBD will be 90% effective in achieving successful biliary decompression, we estimate that 160 patients (80 per study group) would provide a power of at least 85% to detect a 20% absolute difference between study groups on the basis of Fisher’s exact test, with a two-sided significance level of 0.05. To address potential losses to follow-up, the sample size is inflated by 15%. Thus, a total of 184 subjects will be enrolled.

For analysis of the primary endpoint, we will use a chi-square test to compare the proportion of patients achieving a 50% reduction from baseline in serum bilirubin level within 3 weeks in the PTBD and ERC groups using the intention-to-treat (ITT) principle. All randomized subjects will comprise the ITT population. A secondary analysis will explore the potential variability by study site and baseline serum bilirubin on the primary outcome measure. Center differences will be examined by including study site as a covariate in a mixed-effects logistic regression model. Since there will be roughly 25 centers with variable sample sizes, study site will be modeled as a random effect. In addition to the defined ITT analysis sample, a per-protocol sample will be defined as a subset of the ITT sample. This sample will be used for secondary sensitivity analyses of the primary and secondary outcomes. The per-protocol sample will include all randomized subjects that do not have the following protocol deviations: eligibility violation, non-adherence to the randomized treatment, or missing primary outcome. The secondary endpoints will be compared between treatment arms using a chi-square test or *t* test for independent means, as appropriate. There will be no adjustment for multiple comparisons in the pre-specified secondary analyses – including any patient-centered outcomes defined in the planning phase – but all additional exploratory analysis results will be interpreted more conservatively using a significance level of 0.01

Recognizing that the study population may be heterogeneous in ways which could differentially impact the success of ERC vs. PTC, we plan on performing pre-defined exploratory analyses of treatment differences in the primary outcome, adjusting for possible prognostic variables including: (1) age, (2) Body Mass Index (BMI), (3) Charlson Comorbidity Index, (4) presence of proven malignancy, (5) stricture of Bismuth type, (6) presence of significant unilateral lobar atrophy, and (7) recruitment at a center enrolling an average of at least four patients/year. Each covariate will be evaluated individually first in a logistic regression model that includes an interaction effect with the treatment. Interactions will be examined at significance level of 0.15, though the trial may be underpowered to assess such interactions. If significant interaction (statistical and clinical) is concluded, subgroup analyses may be considered. A multi-variable model that includes covariates that contributed significantly as treatment modifiers individually may then be constructed.

## Discussion

The INTERCPT trial will be the only randomized comparison of PTBD vs. ERC as the initial intervention for patients with suspected malignant hilar obstruction. This is a fundamental question in clinical practice that has remained unanswered because of the practical and methodological complexities of such a trial.

To maximize practicality and generalizability, we have adopted a pragmatic study design that includes only one protocol-driven intervention – randomization to PTBD or ERC. All other clinical decisions are deferred to treating physicians as clinically appropriate. We believe that this experimental question is particularly suited for such a pragmatically designed trial because the varying approaches to managing this highly complex disease process between patients and across practice settings constrains protocol standardization and threatens the generalizability of a more traditional randomized controlled trial (RCT) design. Standardizing procedural approach, re-interventions, and medication administration, for example, would not only be impossible to ensure, but would also critically limit external validity because of the tremendous complexity in decision-making and the extreme variability in approaches between providers and institutions.

We appreciate that physicians may select ERC vs. PTBD based on stricture characteristics and other considerations such as the patient’s overall health and goals of care. However, in many practice settings, ERC is typically attempted first because of its perceived advantages. Moreover, stricture characteristics are often unknown beforehand because routine MRI/ magnetic resonance cholangiopancreatography (MRCP) has not been obtained. Fundamentally, the goal of our study is to determine, at the point of care, whether ERC or PTBD is preferable as the initial treatment of patients with suspected MHO, even though stricture characteristics, resectability status, and a definitive diagnosis may not be known – the exact scenario often faced in real-world clinical practice. Furthermore, this study may determine whether a strategy of routine “planning” MRI/MRCP in these cases to guide procedural selection improves clinical outcomes.

We recognize that patients with suspected MHO are often initially managed by gastroenterologists who may be biased in favor of ERC and may prematurely re-divert patients assigned to the PTBD group back to ERC, reducing the likelihood of successful drainage via PTBD. However, this potential bias will be mitigated by the fact that suspected MHO patients are generally managed in multi-disciplinary fashion by a Tumor Board, minimizing the influence of any single provider on the care of a patient. We also recognize that ERC is well established in the United States and Europe as the initial management option for MHO, and thus it is possible that the results of this study would not impact clinic practice. However, we have selected a large enough effect size such that a demonstrated superiority of PTBD would have to be strongly considered in clinical decision-making and guidelines.

The INTERCPT trial will be the first to determine whether PTBD or ERC is the better initial intervention for a patient with suspected MHO, or whether the treatment approach should be individualized based on stricture characteristics or other factors.

### Trial status

This RCT began enrolling patients in October of 2017.

## Additional files

Additional file 1: SPIRIT 2013 Checklist: recommended items to address in a clinical trial protocol and related documents. (DOC 122 kb)

Additional file 2: CONSORT 2010 Checklist of information to include when reporting a randomised trial. (DOC 216 kb)

## Abbreviations

CT: Computed tomography
ERC: Endoscopic retrograde cholangiography
INTERCPT: INTerventional Radiology vs. ERC for Perihilar Tumors
MHO: Malignant biliary obstruction
MRCP: Magnetic resonance cholangiopancreatography
MRI: Magnetic resonance imaging
PTBD: Percutaneous transhepatic biliary drainage
